# Aseptic Sinusitis: A New Phenotype of Immune‐Related Rhinosinusitis in the Era of Immune Checkpoint Inhibitors

**DOI:** 10.1002/ccr3.71599

**Published:** 2025-12-17

**Authors:** Josephine Yalovitser, Kenny Nguyen, Kelly McKenna, Shahid Ahmed, Carolyn Orgain

**Affiliations:** ^1^ Larner College of Medicine at the University of Vermont Burlington Vermont USA; ^2^ Department of Surgery, Division of Otolaryngology University of Vermont Medical Center Burlington Vermont USA; ^3^ Department of Medicine, Division of Hematology and Oncology University of Vermont Medical Center Burlington Vermont USA

**Keywords:** ear nose and throat, immunology, oncology, otolaryngology, pharmacology and pharmacy

## Abstract

We present two novel cases of aseptic sinusitis resulting from PDL‐1 checkpoint inhibitor use. Prompt identification and diagnosis and a multidisciplinary approach with oncology and rheumatology are imperative. High‐dose steroids are preferable to antibiotics or antihistamines due to primary cancer treatment outcomes.

## Graphical abstract

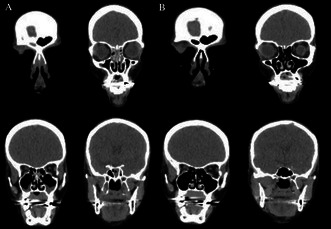

## 1 Introduction

Immune checkpoint inhibitor (ICI) therapies induce the immune system to target malignant cells. While highly effective, they can cause severe and unintended autoimmune reactions called immune‐related adverse events (IRAEs) via the downstream effects of T‐cell activation [1]. Nivolumab and pembrolizumab are PDL‐1 targeted checkpoint inhibitors that have been associated with numerous IRAEs including hepatitis, thyroiditis, dermatitis, and nephritis [2]. As these drugs are beginning to be used more broadly, new IRAE subtypes continue to be discovered. Prompt identification and timely management with corticosteroids is essential, as misdiagnosis may lead to inappropriate antibiotic use and potentially worse oncologic outcomes. We present two cases of IRAEs manifesting as aseptic rhinosinusitis after PDL‐1 checkpoint inhibitor use.

## 2 Case History

Patient 1 is a 64‐year‐old woman with T4bN1M0 nodular melanoma of the left upper back treated with wide local surgical excision of the lesion and a left axillary sentinel lymph node biopsy. Post‐treatment nivolumab was administered monthly for 10 cycles. One month after completing treatment, the patient developed recurrence to a distant cutaneous site which was treated with an injection of Talimogene Laherparepvec (“T‐VEC”), an oncolytic virus that also induces a bystander immune response. She soon developed autoimmune pancreatitis and hepatitis, diagnosed as IRAEs, requiring admission. Nivolumab was discontinued, and she underwent oral steroid therapy (50 mg prednisone taper) with resolution of her pancreatitis and hepatitis. The recurrence was surgically excised.

**FIGURE 1:**
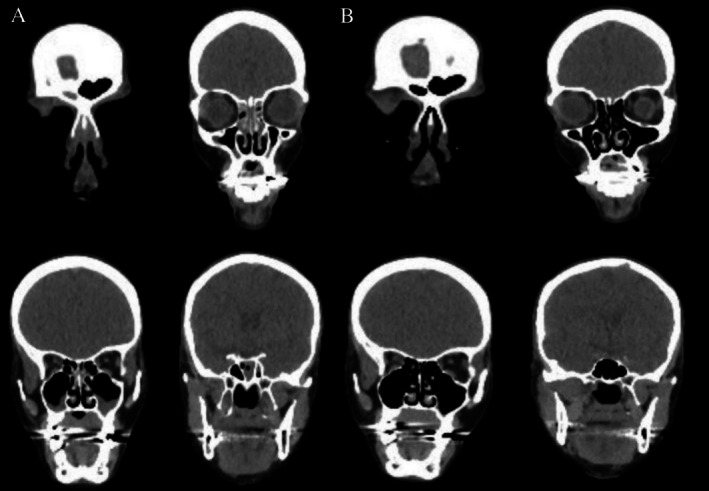
(A–B): Patient 1 CT Imaging of sinuses before (A) and after (B) completing 2 months of steroid therapy for immune‐mediated sinusitis.

The following month, the patient was seen by otolaryngology for anosmia, bilateral nasal obstruction, nasal congestion, and facial pain refractory to daily saline nasal rinses. Her postexcision PET/CT showed near‐pan‐sinus partial opacifications (Figure 1A), along with findings to suggest recent pancreatitis. Nasal endoscopy revealed bilateral middle meatal edema and copious purulent drainage. The culture obtained was aseptic with many neutrophils. The SNOT‐22 score was not completed at this visit.

Patient 2 is a 70‐year‐old woman with T2aN0M1a stage IV nonsmall cell lung adenocarcinoma. She underwent a right craniotomy for dural hemorrhagic metastases and then underwent an initial infusion of 200 mg of pembrolizumab. The patient was switched to selpercatinib due to RET positivity, which resulted in systemic inflammatory response syndrome after the first dose. This medication was discontinued and the patient received 200 mg of pembrolizumab for three cycles and 400 mg of pembrolizumab for an additional 12 cycles. She underwent stereotactic radiation therapy to the right hilum, right lower lobe, and the right supraclavicular lymph nodes. Due to the development of recurrent colitis, pembrolizumab was discontinued. She then received four infliximab infusions with eventual achievement of remission.

**FIGURE 2:**
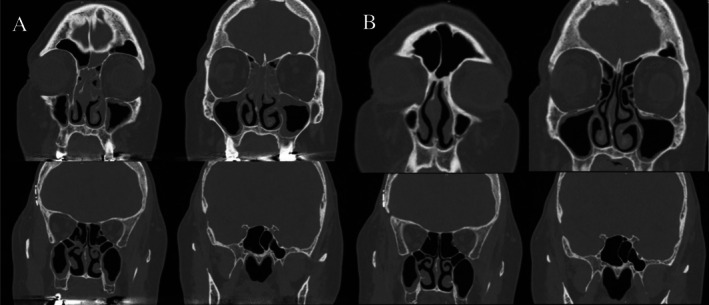
(A‐B): Patient 2 CT Imaging of sinuses before (A) and after (B) completing 6 weeks of steroid therapy for immune‐mediated sinusitis.

Three months after completing immunotherapy, the patient presented to the otolaryngology clinic with a 1‐month history of nasal congestion, postnasal drip, and hyposmia refractory to nasal irrigation and two courses of amoxicillin‐clavulanic acid. CT showed bilateral maxillary sinus mucosal thickening and near‐total bilateral ethmoid opacification; SNOT‐22 score was 54 (Figure 2A). Bacterial culture revealed aseptic sinusitis with many neutrophils. ANCA, Proteinase 3, and Myeloperoxidase antibody screens were negative.

### 2.1 Differential Diagnosis

Symptoms of nasal congestion, postnasal drip, and hyposmia were initially managed with daily saline nasal rinses and empiric antibiotics. However, recent treatment with immune checkpoint inhibitors, combined with aseptic bacterial cultures demonstrating neutrophil predominance, raised concern for an immune‐mediated sinusitis and pointed more toward a diagnosis of autoimmune sinusitis. Although T‐VEC‐related sinus inflammation was also considered for Patient 1, reported adverse events with this therapy are typically limited to flu‐like symptoms, gastrointestinal upset, musculoskeletal pain, headaches, and occasional respiratory effects. Clinical trial data and postmarketing pharmacologic analysis do not identify sinusitis as an associated toxicity, suggesting that T‐VEC‐associated aseptic sinusitis would be exceedingly rare and, to our knowledge, unreported to date.

## 3 Conclusion and Results

Patient 1 was placed on systemic steroids (30 mg prednisone taper over 4 weeks) and daily 0.5 mg budesonide rinses. She reported near‐total symptomatic improvement within days of initiating treatment. Follow‐up imaging 2 months later showed no evidence of sinusitis (Figure 1B), and the steroid rinses were discontinued. SNOT‐22 score was 0 following treatment. The patient has remained free of melanoma, aseptic sinus disease, and other autoimmune conditions for over 18 months and has not received any further immune‐related treatments.

**FIGURE 3:**
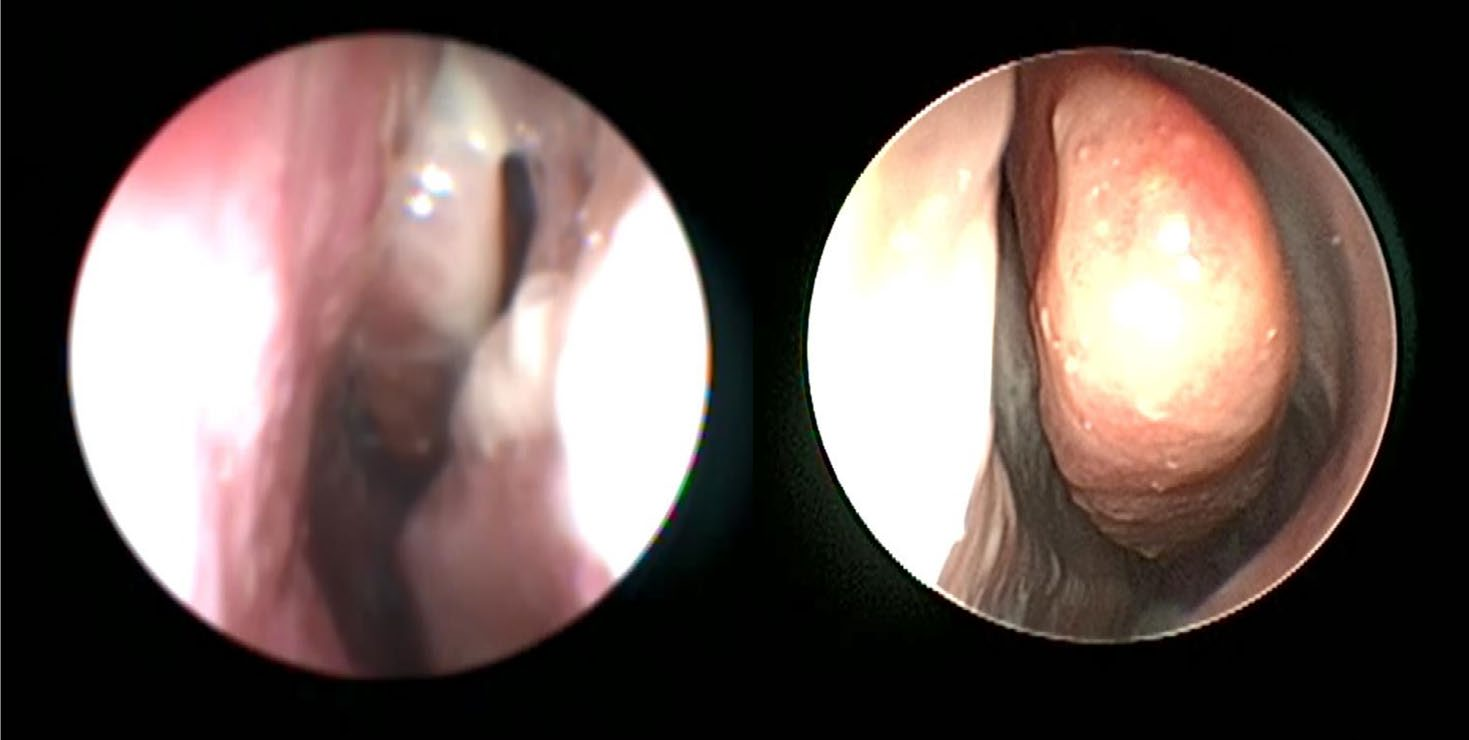
Patient 2 endoscopy of left middle turbinate and middle meatus demonstrating nasal tissue edema and purulent discharge on the left, with resolution of purulent discharge after treatment on the right.

Patient 2 was started on a 30 mg prednisone 4‐week taper and twice‐daily 0.5 mg budesonide saline sinus rinses. After 6 weeks of treatment, she had significant relief in symptoms with resolution of sinusitis on CT and endoscopy (Figures 2B, 3); SNOT‐22 score improved to 24.

## 4 Discussion

Both patients described had experienced multiple IRAEs after treatment with ICIs including aseptic sinusitis, hepatitis, and pancreatitis, which suggests that autoimmune sinusitis tends to co‐occur with other IRAEs. All of these resolved with courses of high‐dose oral steroids and nasal steroid rinses. There are at least two other cases of aseptic sinusitis after the use of ICIs reported in the literature [1]. These previous cases were treated with biologic therapy rather than high‐dose steroids. The leading consensus is that this novel form of sinusitis seen in the setting of ICI therapy is likely the result of autoinflammation induced by nonspecific T‐cell activation [1].

Up to 80% of ICI patients develop IRAEs, for which steroids are the principal treatment [3]. As seen with these patients, IRAEs can also occur after the cessation of treatment and should be suspected for up to a year after stopping immunotherapy. The majority of IRAEs are low‐grade, steroid‐sensitive, and resolve within 6–12 weeks of treatment—especially when steroids are initiated early [3, 4]. Steroids should be given at autoimmune‐equivalent doses, tapered gradually to minimize recurrence. ICIs may be continued alongside steroids for mild IRAEs but should be paused or discontinued in more severe cases. Further studies are needed to investigate the effects of steroid use on ICI efficacy. In steroid‐refractory cases, immune‐modulatory agents such as TNF‐α inhibitors may be considered [4].

In a retrospective review of 108 patients receiving pembrolizumab for metastatic melanoma, 12 patients developed chronic rhinosinusitis symptom(s) an average of 5.2 months after treatment initiation [5]. While management of these patients included steroid nasal sprays and sinus irrigation, the use of oral steroids was limited and pembrolizumab was continued through treatment. Topical triamcinolone in carboxymethylcellulose nasal packing was suggested as a nonsurgical treatment option for refractory cases [5].

The differential diagnosis for aseptic sinusitis in ICI‐treated patients includes allergic sinusitis, which can present with overlapping symptoms such as nasal congestion, rhinorrhea, and sinus pressure. However, allergic sinusitis is typically associated with a history of atopy, seasonal variation, eosinophilia, and elevated serum IgE levels. In contrast, ICI‐related aseptic sinusitis lacks these markers and is more often characterized by concurrent systemic IRAEs, a lack of identifiable infectious pathogens, and responsiveness to immunosuppression rather than antihistamines [6]. Endoscopic findings may also differ, with allergic sinusitis showing mucosal edema and polyp formation, whereas ICI‐induced sinusitis may show more diffuse inflammation without classic allergic features [6].

Given that the majority of IRAEs are treatable and fully reversible, the long‐term sequelae of untreated IRAEs remain unclear. One retrospective study of 496 melanoma patients found variable results in which events resolved or did not resolve without treatment [7]. The majority of these events were mild and dermatologic in nature.

The symptom profile and endoscopic exams of IRAE sinusitis closely mimic bacterial sinusitis but require a drastically different treatment plan. Identification of possible autoimmune sinusitis is of particular importance in patients with stage III or IV melanoma undergoing ICI therapy. These patients can have worse treatment outcomes with increased antibiotic exposure, an effect primarily attributed to a lack of an immune response owing to decreased gut microbial diversity [8]. As such, it is paramount for otolaryngologists to be cautious when prescribing antibiotics for sinusitis in this patient population. Prompt diagnosis of patients with autoimmune aseptic sinusitis due to ICI therapy is necessary for early initiation of appropriate treatments. A multidisciplinary discussion with oncology and rheumatology is encouraged when such cases are suspected.

## Author Contributions

**Josephine Yalovitser:** conceptualization, data curation, resources, writing – original draft, writing – review and editing. **Kenny Nguyen:** data curation, writing – original draft, writing – review and editing. **Kelly McKenna:** conceptualization, investigation, supervision, writing – review and editing. **Shahid Ahmed:** conceptualization, investigation, writing – review and editing. **Carolyn Orgain:** conceptualization, investigation, supervision, writing – review and editing.

## Funding

The authors have nothing to report.

## Consent

The authors of this case report have obtained signed written informed consent from all the patients in this report to publish in accordance with the journal's patient consent policy.

## Conflicts of Interest

The authors declare no conflicts of interest.

## Data Availability

Data available on request from the authors.

## References

[1] E. Dein , W. Sharfman , J. Kim , et al., “Two Cases of Sinusitis Induced by Immune Checkpoint Inhibition,” Journal of Immunotherapy 40, no. 8 (2017): 312–314. 28614096 10.1097/CJI.0000000000000174 PMC5593775

[2] A. R. Almutairi , A. McBride , M. Slack , B. L. Erstad , and I. Abraham , “Potential Immune‐Related Adverse Events Associated With Monotherapy and Combination Therapy of Ipilimumab, Nivolumab, and Pembrolizumab for Advanced Melanoma: A Systematic Review and Meta‐Analysis,” Frontiers in Oncology 10 (2020): 10. 32117745 10.3389/fonc.2020.00091 PMC7033582

[3] J. S. Weber , R. Dummer , V. de Pril , C. Lebbé , F. S. Hodi , and for the MDX010‐20 Investigators , “Patterns of Onset and Resolution of Immune‐Related Adverse Events of Special Interest With Ipilimumab,” Cancer 119, no. 9 (2013): 1675–1682. 23400564 10.1002/cncr.27969

[4] J. M. Michot , C. Bigenwald , S. Champiat , et al., “Immune‐Related Adverse Events With Immune Checkpoint Blockade: A Comprehensive Review,” European Journal of Cancer 54 (2016): 139–148. 26765102 10.1016/j.ejca.2015.11.016

[5] T. Standiford , N. N. Patel , A. Singh , et al., “Pembrolizumab‐Associated Chronic Rhinosinusitis: A New Endotype and Management Considerations,” International Forum of Allergy & Rhinology 13 (2023): 2248–2251. 37317899 10.1002/alr.23213

[6] C. Bachert , B. Marple , R. J. Schlosser , et al., “Adult Chronic Rhinosinusitis,” Nature Reviews. Disease Primers 6, no. 1 (2020): 86. 10.1038/s41572-020-00218-1 33122665

[7] L. Hofmann , A. Forschner , C. Loquai , et al., “Cutaneous, Gastrointestinal, Hepatic, Endocrine, and Renal Side‐Effects of Anti‐PD‐1 Therapy,” European Journal of Cancer 60 (2016): 190–209. 27085692 10.1016/j.ejca.2016.02.025

[8] J. J. Mohiuddin , B. Chu , A. Facciabene , et al., “Association of Antibiotic Exposure With Survival and Toxicity in Patients With Melanoma Receiving Immunotherapy,” JNCI Journal of the National Cancer Institute 113, no. 2 (2020): 162–170. 10.1093/jnci/djaa057 PMC7850522 32294209

